# Does Integrated Care Carry the Gene of Bureaucracy? Lessons from the Case of Québec

**DOI:** 10.5334/ijic.5658

**Published:** 2021-11-08

**Authors:** Yves Couturier, Paul Wankah, Maxime Guillette, Louise Belzile

**Affiliations:** 1 Université de Sherbrooke, Québec, CA; 2 Centre de recherche du CHUS, Québec, CA

**Keywords:** integrated care, bureaucracy, implementation, healthcare reforms

## Abstract

**Introduction:**

Demographic and epidemiological transitions of industralized countries mean health systems have to integrate health and social services to respond to the changing needs of their populations. Efforts to integrate care involve important policy and structural changes. This paper examines whether integration efforts are lost in translation during the bureaucratic appropriation of models, or, in an allegorical way, do they reveal genes of bureaucracy?

**Description:**

Since the 1960s, the health system of Québec has undergone four major structural and progressively integrative transformations, characterized as – modernization, shock of reality, explicit integration, and centralization phases.

**Discussion:**

Although integration efforts progressively transformed Québec’s health and social services system, embedded bureaucracies impeded the realisation of these projects. Notably, inadequate change management strategies and lack of integrated funding models hindered integration efforts. Furthermore, there was variability in government prioritisation and support of different aspects of the model by making some components happen, helping others happen and letting others happen.

**Conclusion:**

Drawing insights from bureaucratic obstacles to integration efforts may improve implementation strategies. This paper highlights important policy and administrative challenges that have to be taken into consideration in improving the implementation of integrated care initiatives in a real-life context.

## Introduction

Industrialized countries are experiencing significant demographic and epidemiological shifts in their populations manifest in increasing proportions of community-dwelling seniors living with multiple chronic diseases – including in Québec, Canada [1,2]. This double transition called for shifts in paradigms in the organisation of health and social services systems [3]. Specifically, industrialised countries must shift from traditional hospital-centric models that focused on acute care towards innovative client-centred models that focus on delivering comprehensive continuum of services for chronic conditions [4]. Primary health care has an important role to play in this paradigm shift. For example, community-dwelling older adult at risk of dependency need primary care approaches from various clinically inter-dependent actors working in public, private and community organisations. The approaches of these actors are based on a wide variety of operating modes and priorities [5]. For the past 40 years, this diversity of logics of action has driven the design and implementation of models of organising health care services that aim to heighten connectivity and coordination of professional practices across organisational boundaries [6] – in other words enhancing integrated health and social services.

Although health systems face significant challenges in implementing integrated care models all over the world, the ideals of efficient, and effective services drive policymakers to lead in the modernization of their health systems. That is why there many conceptual models [7] and public recommendations [8] that guide policy makers in their efforts to reform their health systems. In fact, the idea of integrating health and social services is appealing. Who would want a fragmented or even disintegrated health and social services system?

The conceptual proliferation of integrated care models generated several pilot projects, whose evaluations tend to show the promising potential of integrated care strategies at the local level. Although recommendations supporting the implementation of integrated care remain optimistic, converging meta-analysis question the capacity for integrated care models to realise expected outcomes in real-life settings [9,10]. There are substantial challenges to scale up complex local innovations to the level of health and social service systems.

There are several reasons why scaling up complex innovations like integrated care models are particularly challenging. First, organisational actors are known to resist changes in practices promoted by health care reforms (*path dependency*) [11]. Second, the health care system is characterised by structural inertia – due to the distribution of power between multiple actors [12]. Furthermore, difficulties in change management while scaling up these promising innovations may also explain mixed outcomes of integrated care interventions. But can it also be that the very nature of integrated care contains conditions for its ineffectiveness in a real-life context? A kind of bureaucratic gene, allegorically similar to an autoimmune condition of integrated care that empties integration efforts of their vitality. This paper examines this hypothesis through an analysis of integrated care reforms of the province of Québec.

## Methods statement

This paper is based on 20 years of qualitative research on the implementation of very proactive public policies aimed at integrating health and social services in Québec. The first author of this article held a Canada research chair in integrated care from 2009 to 2019 [13]. The chair’s team carried out thousands of hours of observations and hundreds of interviews with users and their families/informal caregivers, managers, various clinicians (including case managers) and policy makers. Our results do not represent an evaluation of the clinical effects of integrated care reforms in Québec. Instead, we sought to better understand the main determinants of the shift of Québec’s health system towards greater integration of health and social services.

## Description of integrated care policy reforms in Québec

In Canada, each provincial government has the constitutional responsibility for administering its health system. Québec is the only French-speaking Canadian province, with 8.5 million inhabitants. Since the creation of its health and social services system in 1966, four extensive structural reforms have shaped the organisation of health services. Each reform has respectively advanced the provincial health system towards greater integration of health and social services. These structural and integrative reforms of the health system represent four overarching phases framed as **modernization, shock of reality, explicit integration**, and **centralization**.

### Modernization phase

The first period of health system reforms (1966–1980), framed as a *modernization phase*, was characterized by the decline of family and charitable models of organising health services that was a key feature of Catholic societies like Québec [14]. This essentially charitable and asylum model posed many challenges in terms of performance and quality of services. The state was powerless in its capacity to regulate health care practices. By returning clients that were previously institutionalized back to their communities, the deinstitutionalization movement that began at the time was, coincidentally, the precursor of current integrated care. While English-speaking Canada, the United Kingdom or the United States had experienced significant modernization of their health system at the end of the Second World War, in Québec, the late shift from the asylum model had the unexpected positive effect of the conception of a very modern health and social services system for the time [15]. Drawing from the best international experiences, Québec’s health system was designed in the early 70s in a more integrated form than other Bismarckian models (e.g. Germany and France) or private systems (e.g. the United States). The most important aspect of this first reform is undoubtedly the creation of a Ministry of Health AND Social Services (MHSS) that explicitly linked the health care and social services sectors. As of date, few countries and Canadian provinces have closely integrated both essential sectors of their health systems. In other words, Québec’s health system was created from a modernist beveridgian concept of integrating health and social services under the same governance structure [14]. At the same time, the Québec Health Insurance fund was created as the sole payer for medical services, mainly by a fee-for-services payment model [16]. Most public non-medical services were financed from taxation, by indexation of historical budgets allocated to organisations [17]. The system was hierarchized into three levels of services (primary, secondary, and tertiary), centred around interprofessional and territorial arrangements of public primary care networks. From an organizational perspective, this reform aimed at creating 300 independent organizations, that were linked to nearly 1000 points of services. These organisations were distributed according to a logic of local territories. At best, only two-thirds of those organizations were effectively established.

### Shock of reality phase

The second period of health system reforms (1980–2003), framed as the *shock of reality phase*, was characterized by increasing awareness of significant gaps in the implementation of activities that were planned during the first period of reforms. Some reasons why the modernization policy reforms were partially implemented include recurring insufficiency of resources to complete the reforms (e.g. territorial coverage was never completed), resistance of physicians to integrate public primary care organizations, and corporatist resistance of unions and professional orders [14]. In order to address these problems, government regionalized the health system, including relative democratization of territorial governance of health care organizations. In addition, government promoted population-based approaches of organizing the health system. Regionalization should have allowed a gradual devolution of powers from the ministry to regional boards mandated to ensure that health services met the needs of their local populations [14]. This approach helped to broaden the framework of public intervention by promoting surveillance activities, community development, prevention and health promotion, etc. This second reform also promoted the relative integration of non-profit organizations into the framework of public interventions.

In the 1990s, there was a major crisis due to difficulties accessing hospital services – a characteristic of most underfunded beveridgian models. This crisis fueled public debates on the best way to use hospitals. Specifically, hospitals were considered as costly resources, especially for patients with chronic conditions whose needs could have been better met at home/community, such as seniors with reduced functional autonomy or people with a advanced mental health conditions [18]. This crisis revealed the effects of the partial implementation of previous policy reforms, and hastened the introduction of an “ambulatory care” model [16], that prioritized health and social care interventions in the community rather than in the hospital.

The idea of addressing aforementioned health system challenges by extending health and social services into the community drove the development of local innovations and research. This period of innovation was largely inspired by early conceptual works explicitly focused on integrated care (e.g. Kodner [19]). Two notable integrated care experiments were carried out in Québec – the SIPA research program (in French : *Services intégrés pour personnes aînées fragiles*) [20] that designed, implemented and evaluated a full integrated model, and the PRISMA research program (in French: *Programme de recherche sur l’intégration des services de maintien de l’autonomie*) [21] that designed, implemented and evaluated a coordination-type integrated care model. These seminal studies greatly informed the thinking of policy makers and explicitly influenced the public policy agenda for integrated care in Québec.

### Explicit integration phase

The third reform period (2003–2014) is framed as the *explicit integration phase*. Encouraged by positive results of pilot-projects in Québec, and based on international conceptual models of integrated care, this reform proposed a major structural reorganization in 2004. For the first time, the MHSS explicitly introduced the principle of integrated care into the design of its health and social services system. Integrated care was presented at the time as a means of accomplishing the shift towards ambulatory care and countering the risks of fragmentation of services. One of the central reasons for this reform that was to improve the usage of hospital resources. Furthermore, the policy reform also aimed at reducing the provincial budget deficit, containing the continuous increase in health care expenditure, and addressing difficult access to services [22]. The MHSS affirmed that:

Integrated care, supported by an appropriate management method, will make it possible to provide a better response to the needs of people by limiting barriers between organisations and professionals. Integrated care is based on the commitment and accountability of clinicians and managers to the population, and on the meanings of functional referral and follow-up mechanisms. More decentralized and flexible organizations, with responsibility for achieving the targeted results, will be created to implement this orientation (translated from the official text) [23].

This integration reform led to the creation of Health and Social Services Centers (HSSC), through administrative mergers of public primary care services, public long-term care services, and acute care hospitals [16]. Hence, 94 HSSC organised and provided health and social services for the entire population – each HSSC served about 100,000 people. Furthermore, HSSCs were responsible for establishing a permanent inter-organizational table between various public, private and community agencies of their territory, called Local Health Networks (LHN) [24]. The HSSC were attributed a population responsibility mandate [25]. According to this integration perspective, the primary responsibility of the health and social services system was to provide services tailored to the needs of their local populations. This included clinical assessment approaches allowing to co-design an intervention plan that integrates all the resources required to meet the needs of clients. For the elderly population, for example, the MHSS implemented a standardized clinical assessment tool (*Outil d’Évaluation Multi clientèle* – OEMC) and a case mix classification system (*Système d’Évaluation de l’Autonomie Fonctionnelle* – SMAF) to determine the needs profile of each client [26]. These profiles are groups of users who present similarities in terms of their socio-sanitary needs grouped into five categories (1. Activities of Daily Living; 2. Mobility; 3. Communication; 4. Mental Functions; 5. Home Living Activities), that were matched to a package of services [27]. These tools (among others) were used by a case manager who was responsible for the professional care coordination of a client needs. An interdisciplinary care plan was co-created with the case manager to include the needs and values of the client. The MHSS and HSSCs use data from care plans to document population needs, and to measure the gap between population needs and services provided. This integrated care model also used a single entry point to direct clients to appropriate services, as well as a computerized clinical record to connect providers in the continuum of care.

In addition to the creation of HSSC, the MHSS concomitantly supported the creation of family medicine groups (FMG). These new organizations generally consisted of 6 to 10 family physicians, providing primary medical care to enrolled patients (1000 to 2200 per full-time family physician) [28]. FMGs were supported by nurses that were under the administrative and clinical responsibility of the HSSC [28]. The implementation of FMGs aimed to facilitate access for clients with chronic conditions to a community-based family physician in order to reduce the number of avoidable hospitalizations [29]. In this light, the creation of FMGs could be viewed as extending the continuum of services for priority clients across organisational boundaries from the public sector (HSSCs) to privately owned medical groups (FMGs).

Overall, this reform consolidated the transition from a system with several hundred independent public organizations to 183 (to which must be added more than 300 FMGs) for the entire territory [30]. These public organizations were coordinated by 18 regional agencies. The creation of HSSCs was accompanied by increased demand for accountability by the ministry [31]. Thus, ministerial desire to increase the autonomy of public health care organisations was strongly regulated and supervised. This represented an evolution in managerial philosophy that focused on performance measurement.

### Centralization phase

The last period of reform, framed as the *centralization phase* [32], was implemented in 2015. It consisted of a major shift in the governance of the health system, by concentrating decision-making power at the ministerial level [33]. Specifically, the ministry abolished Regional Health Authorities, and created Integrated Health and Social Services Centers (*Centres Intégrés de Santé et Services Sociaux* – IHSSC), some of which hold an academic mission (*Centres Intégrés Universitaires de Santé et Services Sociaux*) [14]. By creating these new organizations, the ministry also abolished local governance of health care. By including the term “integrated” to the name of IHSSCs, the ministry explicitly defined their identity. In fact, IHSSCs were created by merging – over an even larger territory – neighbouring HSSCs, and specialized second-line organizations, such as youth protection centers and rehabilitation centers. IHSSCs effectively integrated all public health and social services missions under a single governance structure [14]. Following this centralization reform, Québec only had 34 public health and social services organisations in its territory (each IHSSC served a population of about 250,000 people). This reform occurred concomitantly with an intensification of the organization of medical practice [33]. For example, government supported FMGs with additional non-medical professional resources (e.g., social workers), under the administrative and clinical authority of IHSSCs [34]. None of these reforms modified the funding model of Québec’s health system.

## Recurring difficulties despite progress in the integration of services

We define bureaucracy as the result of a process of standardizing functioning rules of an organization or a system [35], and we use, the terms «gene» as an allegory. Our analysis of the “bureaucracy gene” hypothesis will focus on the last two policy reforms because they explicitly aimed at integrating services. Although there was no formal evaluation of the outcomes of integration efforts [36], these two reforms have been the subject of multiple partial analysis and discussions, most often without these opinions being based on robust empirical data. Nevertheless, there is strong convergence around a feeling of disenchantment, that may be directly attributed to the nature of integrated care.

Some documented impacts of these policy reforms included centralizing decision-making powers at the ministerial level [32], increasing the size of public health care organizations [37], protocolizing professional practices [38], rationalizing the supply of services and transformation of the management philosophy towards a management for value type philosophy [39], de-democratizing governance, among others, without clear added value at the clinical level [22]. These findings are all vectors of bureaucratization. They could perhaps be worthwhile if they produced clear clinical effects in terms of improving access to services, reducing avoidable hospitalizations, or increasing prevention, etc., as shown in the PRISMA and SIPA experimental projects. However, nothing is certain about the outcomes of these system-wide reforms. After nearly 20 years of explicit integration reforms, access to services remains difficult, the hospital still occupies a central place in the health system, the cost of health is still increasing, etc. [40]. In addition, Québec has been very hard hit by COVID-19 (1201 deaths per million inhabitants as of February 2021), especially among seniors with advanced loss of functional autonomy – those who were mostly targeted by integration reforms.

Efforts to integrate services in Québec led to mergers that increased the size of organizations and produced a much more bureaucratic mode of management at the operational level. As a result of these reforms, procurement, human resources, union accreditations, and clinical management, among other dimensions, are occurring on a much larger scale than ever before. There are two main negative effects of these reform movements:

- Increasingly distant relationships between organizations mandated to provide health services and their communities means the integrated organization cuts itself off from one of the aims of the integration project; specifically, to ensure coherent actions of stakeholders of various health and social services organisations in a local area.
- Managerial forces folding back to the needs of their respective organizations due to intense efforts needed to complete the mergers of various public health and social services organisations. It takes at least a decade to complete such deep reforms, and to (perhaps) achieve positive clinical effects. The cycle of major health policy reforms in Québec was carried out approximately every decade. Thus, managers were constantly mobilized for the internal management of structural and intra-organizational effects of reforms, at the expense of advancing good clinical integration For example, managers focused on harmonizing the norms for various parking lots rather than implementing real clinical changes. Those immense managerial efforts had no clear impact on the clinical efficiency of services. All actors involved in the reforms were disenchanted, including senior management.

These reforms also reintroduced politics in clinical activities – by abolishing important intermediary bodies (regional health authorities) that had the role of translating ministerial priorities into operational activities – in the name of more efficient governance, less sensitive to the influences of various local stakeholders. While this end of control can be considered bureaucratically legitimate, in reality, politics is not a factor of stability or predictability. Without an intermediary, the pace of politics is changing the pace of provider organizations, with the bureaucratic force losing its few positive assets because of its greater submission to politics.

The unfortunate example of COVID-19 clearly illustrates these shortcomings. While efforts to integrate services in Québec have notably focused on the continuum of services for seniors, the MHSS focused all its initial efforts during the first months of the pandemic on hospitals, thus revealing the hospital-centrist inclination of politics. Three decades of efforts to establish an integrated continuum of services for seniors with loss of functional autonomy do not appear to have influenced the management of the pandemic.

## When legislating is not doing

The gap between the promises of integrated care and clinical reality is mostly rooted in the Taylorist postulate of policy makers asserting that good public policy should automatically engage the expected behaviors of the people targeted by the change, except malice or incompetence on their part. This gap has taken at least four forms in the case of Québec:

1. None of the four reforms has been completed according to the plan initially designed by policy makers.
2. None of these reforms has been supported by sustained change management strategy.
3. None of the last three reforms proposed to revise the funding model established by the first policy reform. Thus, the funding model is still not integrative.
4. None of the reforms have been rigorously evaluated for their clinical effects, which has hampered the ability to continuously improve the innovation process.

To illustrate this last point, we provide a summary (in ***Table 1***) of the essential components of Québec’s integrated care model for older adults with loss of functional autonomy. We analyzed the quality of their implementation strategy based on the three approaches of change management suggested by Greenhalgh et al [41]. ***Let it happen*** is a passive approach where innovations are disseminated without much support, ***Help it happen*** is an approach where innovations are disseminated with some support, and ***Make it happen*** is an approach where innovations are disseminated with sustained support – in line with this approach, innovations are often mandated.

**Table 1:** State of implementation of components of integrated care for older adults.


KEY COMPONENTS	STATE OF IMPLANTATION 17 YEARS AFTER THE 2004 REFORM	CHANGE MANAGEMENT APPROACH

**Permanent concertation mechanism with regional partners and population responsibility**	Local health networks (LHN) were implemented almost everywhere, but their operations vary a lot. Most LHNs work on small-scale interventions. The 2015 reform pushed concertation mechanisms to a minor role. Integrated governance within IHSSCs has in fact replaced this concertation mechanism in the minds of managers and policy makers.	Let it happen

**Single access point**	Established everywhere, except for FMG clients and all private organizations.	Make it happen

**Standardized evaluation tool**	Established everywhere since 2013, except for FMG clients and all private organizations.	Make it happen

**Case classification system**	Established everywhere since 2013, except for FMG clients and all private organizations.	Make it happen

**Computerized clinical record**	Partial implantation.	Help it happen

**Individualised service plan**	Clinically insignificant, in particular due to a delay in computerization, and the lack of reform of the funding model.	Let it happen

**Case management**	Partial implementation, experiencing many failures, the guideline was not published until 2015.	Let it happen

**Integrated governance**	Merged governance accomplished, but many clinical implementation issues	Make it happen

**Integration of physicians**	Organizational progress through the creation of FMGs, but the medical sector remains very person-dependent, and access has not been significantly improved for priority clienteles (mental health, chronic diseases and loss of functional autonomy).	Help it happen


This analysis shows key components of integrated care from the perspective of the ministry. *Make it happen-type* efforts enabled the MHSS to increase its capacity to lead the health and social services system. To put it bluntly, integrating services has proven most useful in achieving administrative mergers and increasing strategic managers’ control over service delivery. On the other hand, the most adaptive components – all clinical components of integration – such as case management or individualized service plans, were driven by *let it* or *help it happen* change management approaches. The gap in change management strategies by component therefore shows that the reform movement was powerful in terms of managerial processes, much less in terms of clinical effects. The interface components (Local Health Networks and case management) between the two types of processes have been better implemented, but with little organizational effect. The picture would probably be even more disappointing if we considered the clinical effects of these reforms (empowerment, prevention, avoidable hospitalization, etc.).

The intensive managerial efforts to implement these great structural reforms even slowed down the progress of clinical integration at the local level. Our reserved judgement on the clinical dimension of integration could be mitigated if we consider that clinical change is often the last dimension to be achieved, and time will ultimately do its work. This analysis may be legitimate, but it is questioned by the analysis of reforms that show the concomitance of official reforms with a conceptually positive aim – here integrated care – but in fact hiding other reforms pursuing other ends; in this case the implementation of a managerial philosophy inspired by a technocratic version of the *management for value approach* [42]. The locus of innovation is shifted from integrated care, as a systemic adaptation device to the increasing prevalence of chronic conditions, to managerial control of public spending.

Overall, the remarkable capacity of the government of Québec to produce extensive structural changes, of a magnitude that has few international comparisons, was not a sufficient condition for bringing about beneficial effects at the clinical level of integration. Rather, the reforms had bureaucratizing effects that were not always negative. These reforms created favorable conditions for functional, sometimes even professional, changes. For example, managers had more governance capacity to act on contradictory practices within a continuum of services, such as inappropriate duration of hospitalizations for people waiting for home care. These changes are potentially favorable for integrated care, but they unfold so slowly that their meaning is lost in the overall change efforts.

Was integrated care only a rhetoric from policy makers to justify mergers or, were mergers the structural condition for slow but nonetheless real implementation of integrated care? We believe the answer lies between these two positions.

Although the creation of IHSSC enabled the MHSS to increase their capacity to control – and this increased capacity to control has many bureaucratizing effects – it is nevertheless part of an overall integration perspective. Furthermore, there have been positive advancements in functional integration. For example, the creation of service continuums for vulnerable clients. These continuums are currently imperfect, do not achieve all the expected clinical effects, and have taken too long to form – but they do exist and structure the ongoing work of integrating care at the clinical level.

Nonetheless, the creation of the IHSSC is clearly underpinned by a political agenda seeking to impose a managerial and quasi-taylorist conception of the operations of the health and social services system, allowing the ministry to increase their power over local actors. In addition, it has many adverse side effects, including the bureaucratization of relations between local actors, increasingly disconnecting decision-making from local dynamics, the reduction of managerial energy that was directed at achieving mergers, poor local adaptivity, loss of sense of work by professionals, etc. Conceptual models of integrated care have therefore provided an argument for legitimizing takeover, while creating structural conditions that are in principle useful for integrated care.

In fact, this approach of integrating care through structural efforts postulates, even hopes, that positive clinical effects will ultimately emerge. Even if it takes some time to emerge. At worst, positive clinical effects may be of lesser importance as compared to the intense managerial efforts that we had previously mentioned.

The fundamental translation of conceptual models of integrated care by policy makers is related to their political utility in giving meaning, or a clinical appearance, to the often too general principles of management for value [42]. With this conceptual and rhetorical apparatus, policy makers made three fundamental adaptations of the integrated care conceptual models.

The first adaptation was by decoupling the components of integrated care models that were developed by their designers as a coherent whole. Policy makers therefore drew integrated care components closest to their political agenda from various conceptual models and compelled their implementation. In doing so, they set out general conditions for implementing other more clinical components of integration; but left local goodwill to implement them.

The second adaptation was by refusing to act on certain structural determinants (such as funding models) and certain laws characterized by classic inertial forces; for example, constraining interprofessional collaboration (e.g.: circulation of information between professionals). While the state has shown an extremely strong will to carry out major mergers of organizations, this voluntarism has been timid for other components.

The last adaptation was the slow implementation of essential components of conceptual models that experimentation positioned as critical to effective clinical integrated care (e.g. case management). It has the effect of allowing the necessary interdependence of the various components of integrated care to be lost over time.

The quality of implementation processes and change management strategies are frequently raised to explain the gap between the expectations of conceptual models and reality. Although policy reforms explicitly aimed at integrating care in Québec, parallel or hidden reforms a shift towards a governance model requiring accountability for the more tangible aspects of care (e.g. number of interventions rather than empowering effects of interventions) hindered implementation efforts. Hence, the founding principles of integrated care (e.g. empowerment of users, prevention of avoidable health conditions) were of minor importance, then emptied of their meaning for clinicians and local managers as was the case with case management. Likewise, lack of clear definitions of the roles and responsibilities of various stakeholders, lack of definition of the expected effects and a lack of support measures for implementation may partially explain the results of the analysis. For example, case management guidelines were published by government in 2017, almost 13 years after the 2004 reforms, and without any serious implementation plan – till date. For this essential clinical component, varying understandings of its guiding principles at the local level led to the deployment of very different approaches and solutions from one health area to the other, to the point of rendering case management meaningless in many contexts. This may be due to the uncoordinated implementation of certain components, especially the more clinical ones, such as individualized service plans [43].

## Does integrated care contain the gene of bureaucracy?

In the conclusion of an open letter published in 2016 about the last integrated care reforms in Québec, the famous Henry Mintzberg proposed this solution, in a satirical tone:

I have a terrific idea. Do we really need all those government ministers? Health, Justice, Finance, Education, Culture, Agriculture, etc. Let’s try something, just in case it might work. Agglomerate all these departments, and have the premier — he is, after all, the prime minister — run the whole show himself. Try a dose of his own medicine. Think of how much more money we could save [44].

Integrated care spontaneously pleases the mind of the bureaucrat, sensitive by nature to rules, predictability, consistency, and resistant to the singular, the dynamic, and therefore the clinical. Perhaps he could find Mintzberg’s joke admissible! We do not believe, however, that integrated care, such as the allegory of the soapy board, inexorably pushes us towards integrative bureaucracy. In addition to the fact that even a critical analysis of Québec case shows advances at the structural level that are presented in conceptual models of integrated care [45], it is possible to contain bureaucratic temptation by reaffirming certain essential principles:

- Integrated care must remain above all a clinical project.
- Structural changes required for its implementation must be rigorously evaluated in relation to their clinical effects.
- The relationship between provider organizations and the communities they serve must remain close, and therefore anchored in local realities.
- Due to its complexity, scaling-up integration pilot projects at the health system level must be subject to change management at the level of the integration project.
- The clinical or adaptive components (case management, concertation, individualised service plan, etc.) must be the subject of sustained change management efforts in order to preserve the overall meaning of the reforms. Managerial components should thus be at the service of the clinical and not the other way around.

## Conclusion

The Québec experience shows that mergers of organizations do not always have the expected clinical effects, due to the use of integrated care arguments for the purpose of implementing managerial approaches. Mergers nevertheless create structural conditions that are favorable for certain dimensions of integration, but at the cost of potentially distancing relationship with local communities and clinical ends. Serious implementation of case management and local health networks, as well as the sub-territorialization of certain healthcare teams, such as those working in home care, can partly contain the harmful effects of mergers.

Along the lines of the quadruple-aim, Québec’s major reforms increased the capacity for senior managers to master the rules structuring its health care system. However, this was done at the cost of loss of capacity by field managers. While the reforms seem to be sincerely interested in the health of the population, it is impossible at this time to know whether the reforms have had any real clinical outcomes. Finally, our work shows that the experience of patient care such as the well-being of care teams was not improved by 20 years of efforts to integrate care.

What remains at stake is the intensity of managerial efforts required by mergers and its effect on the ability to effectuate changes required to achieve the expected clinical effects of the integrated care. Does this invalidate the “full integration models” in favor of the “coordination models”? Perhaps. We think, however, that above all the real debate should focus on identifying what should be fully integrated (sometimes taking the form of mergers) and what requires a more flexible, more bottom-up, more coordinated approach – from the perspective of clinical efficacy. This comes down to questioning the scale of the geographic area that the integrated organisation serves. Certain services (e.g. rehabilitation) may be provided at a larger scale than others. It may be important to determine what services are more effective at a scale that is closer to the communities they serve.

Does the top-down and quasi-taylorist approach adopted by Québec explain this state of affairs? Or is it rather integrated care that contains within it the gene for such a takeover of managerial power over the professionals? Be that as it may, it is not a question of decrying the Québec example by affirming that it demonstrates any superiority over bottom-up models of integrated care that are very fashionable in recent years all over the world. A rigorous analysis of the outcomes of bottom up models should also shows different weaknesses, in particular due to the maintenance of structural determinants of fragmented of services (maintaining two ministries, health and social services, funding methods, information infrastructure, etc.). A comparative analysis with other jurisdictions (such as France or Germany) would probably shows that a totally bottom-up approach valued in these contexts also has its own limits. Thus, it seems too easy to argue that structural mergers and the top-down approach deployed in Québec alone explain the timid judgment that we are making here on the state of integrated care in this jurisdiction in terms of outcomes.

What is rather in question in the Québec case is the frequency and speed of structural changes as compared to the slow clinical changes. It evokes the naive hope that structural changes will magically initiate the desired changes in clinical practices, without close and lasting support for change, combined with a sustained strategy of sense-making. To use the genetic analogy, we conclude that integrated care models do not contain the gene for bureaucracy, but that they contain conditions for expressing this gene in any policy maker. How then to guard against it? By favoring a strictly bottom-up approach allowing integrated care to be part of a succession of small temporary and local changes that have little impact at a bigger scale? No, because in our opinion it is not a question of choosing between bottom-up or top-down change management, but rather of finding the right balance between the two, according to the specific needs of each health system. Moreover, the (relative) disappointment of the Québec experience shows that the solution to containing the risk of drifting towards bureaucratization consists above all in attaching any integrated care reform, top down as well as bottom up, to a rigorous evaluation of its clinical effects, the more relevant effects.
